# The Role of Osteoclast Energy Metabolism in the Occurrence and Development of Osteoporosis

**DOI:** 10.3389/fendo.2021.675385

**Published:** 2021-05-12

**Authors:** Wacili Da, Lin Tao, Yue Zhu

**Affiliations:** Department of Orthopedics, The First Hospital of China Medical University, Shenyang, China

**Keywords:** osteoclasts, osteoporosis, energy metabolism, bone homeostasis, bone resorption

## Abstract

In recent decades, the mechanism underlying bone metabolic disorders based on energy metabolism has been heavily researched. Bone resorption by osteoclasts plays an important role in the occurrence and development of osteoporosis. However, the mechanism underlying the osteoclast energy metabolism disorder that interferes with bone homeostasis has not been determined. Bone resorption by osteoclasts is a process that consumes large amounts of adenosine triphosphate (ATP) produced by glycolysis and oxidative phosphorylation. In addition to glucose, fatty acids and amino acids can also be used as substrates to produce energy through oxidative phosphorylation. In this review, we summarize and analyze the energy-based phenotypic changes, epigenetic regulation, and coupling with systemic energy metabolism of osteoclasts during the development and progression of osteoporosis. At the same time, we propose a hypothesis, the compensatory recovery mechanism (involving the balance between osteoclast survival and functional activation), which may provide a new approach for the treatment of osteoporosis.

## Introduction

The human skeleton is in a state of constant renewal, in which new bones are generated and old bones are degraded. At a young age, the body produces new bones faster than it degrades old bones (resulting in net bone accumulation), and bone mass increases. After the age of 20, this process slows down, and most people reach their peak bone mass by the age of 30 (1, 2). Osteoblasts and osteoclasts (OCs) are the main factors involved in bone remodeling in the bone microenvironment. The balance of osteogenic and lipogenic differentiation of bone marrow mesenchymal stem cells controls the osteoblasts involved in the formation of new bone mass (3). Osteoclasts involved in bone resorption are derived from monocyte/macrophage lineage cells, which are fused to form multicellular giant cells that are capable of bone resorption (4). Osteoporosis (OP) is a chronic, systemic endocrine and metabolic disorder, whether it is primary osteoporosis due to aging or sex hormone deficiency or secondary osteoporosis due to hyperthyroidism, diabetes, obesity, Cushing’s syndrome, anorexia, rheumatoid arthritis and drug effects. The underlying mechanism is the imbalance of bone remodeling in which the loss of bone mass occurs faster than bone production (5–11). Osteoporosis and related fractures have become a major public health problem and have substantially increased health care costs. Research on the pathological mechanism of osteoporosis will help to reduce the high costs associated with osteoporosis and improve the quality of life of elderly individuals.

The balance of bone remodeling is dynamic and vulnerable to external stimuli, including energy metabolism substrates, hormones and growth factors. Both bone formation by osteoblasts and bone resorption by osteoclasts depend strongly on energy expenditure. In recent years, the pathogenesis and treatment of various diseases in the body based on energy metabolism, such as tumors, cardiovascular diseases and nervous system diseases (12–15), has been a hot topic. Osteoporosis is also a disorder of systemic energy metabolism and glucose and lipid metabolism characterized by an abnormal fatty acid distribution and amino acid content, all of which are closely related to the occurrence and development of osteoporosis (8, 16–18). Focusing on the bone microenvironment, the specific energy metabolism disorder of osteoblasts and osteoclasts is the key to pathogenesis. The energy produced by a cell is mainly dependent on glucose, and glycolysis (cytoplasm), the tricarboxylic acid cycle (TCA cycle) and oxidative phosphorylation (OXPHOS, mitochondria) are the fundamental pathways that produce adenosine triphosphate (ATP, the most important high-energy chemical in the body) (19). Here, we must expound on the basic process of cell energy metabolism. ATP is produced by the decomposition of glucose in nine continuous glycolysis reactions, the terminal product pyruvate is converted to acetyl coenzyme A by pyruvate dehydrogenase under aerobic conditions to activate the TCA cycle, and it is also converted to lactate by lactate dehydrogenase under anoxic conditions (19). In some cases, glucose is converted to lactate under aerobic conditions through a process known as aerobic glycolysis (also known as the Warburg effect) (20). In addition to glucose, fatty acids and amino acids can also be used as substrates to produce energy through oxidative phosphorylation (21–23). Free fatty acids derived from dietary fat or from triglyceride hydrolysis in fat cells are another effective substrate for energy production. The oxidation of fat produces more than twice as much energy as the oxidation of the same weight of carbohydrates or proteins (21). Certain amino acids can also be used as substrates for glycolysis and/or further generation of ATP through the TCA cycle (23).

Mitochondria are the key organelles for energy metabolism, and their outer membrane contains porins with high permeability; the inner membrane is highly opaque and folds inward to form cristae containing energy conversion-related proteins (electron transport chain, ATP synthase, and endometrial transport protein) (24, 25). Meanwhile, the production of oxygen free radicals in mitochondria is closely related to cellular signal transduction, transmembrane ion transport and electrolyte homeostasis (26, 27). The carbon-hydrogen bonds in the molecular structure of energy substances such as glucose, amino acids and fatty acids contain chemical energy; in the process of oxidation, the carbon-hydrogen bond breaks and produces CO_2_ and H_2_O while releasing the stored energy. The balance of the chemical energy within the cell regulates the cascade amplification mechanism of many molecular signals within the cell, thus controlling the process of gene transcription and translation and finally controlling the phenotype of the cell (28, 29). Bone is composed of minerals such as calcium phosphate and calcium carbonate calcium citrate, as well as organic ingredients such as collagen matrix (30). Osteoclasts differentiate from the original mononuclear progenitor cells and then fuse to form multinucleated cells, a process that requires metabolic reprogramming to maintain biosynthetic substrates and the energy supply (31). Afterwards, mature osteoclasts adhere to the bone surface through αvβ integrin and form an F-actin-sealed zone to absorb the bone matrix, while the acidic environment in the bone resorption region relies on carbonic anhydrase II to generate HCO^-^ and H^+^, which passes through the folds of osteoclasts through vacuolar H^+^ adenosine triphosphatase (V-ATPase) to the absorption pit (32, 33). Local acidosis in the absorption chamber erodes inorganic minerals, leading to the exposure of the organic collagen matrix to proteolytic enzymes, including collagenase, cathepsin K (CTSK) and matrix metalloproteinases (MMPs) (34, 35). In addition, the migration of osteoclasts along the bone surface contributes to the continuity of bone resorption, which is achieved through the dynamic rearrangement of the actin and microtubule cytoskeleton and requires excessive ATP consumption (29, 36). Furthermore, mitochondria regulate bone resorptive activity and OC survival by balancing ATP levels and uses in the cell. Mature OCs rich in mitochondrial DNA release ATP from mitochondria stores into the cytoplasm, and subsequent ATP consumption leads to increased bone resorption (37). Osteoporosis results from excessive bone resorption, which is controlled by two pathways. First, the number of osteoclasts involved in bone resorption on the bone surface is abnormally increased (including increased osteoclast differentiation, excessive proliferation, autophagy dysfunction, and reduced apoptosis); second, the increased osteoclast activity also refers to the enhanced bone resorptive activity (including increased secretion of enzymes such as CTSK, MMP-9 and MMP-13, increased local acidity, and sustained production of ATP). However, after reviewing the studies on the energy metabolism of osteoporosis published in recent decades, few studies have examined the pathogenesis of abnormal energy metabolism in osteoclasts compared with osteoblasts. Therefore, previous studies assessing the energy metabolism of osteoclasts and osteoporosis must be analyzed, the instructive results must be summarized and the existing research defects must be clarified to provide directions for future studies.

## Phenotypic Changes in Osteoclasts During the Development of Osteoporosis Based on Energy Metabolism

### Differentiation

**Figure 1:**
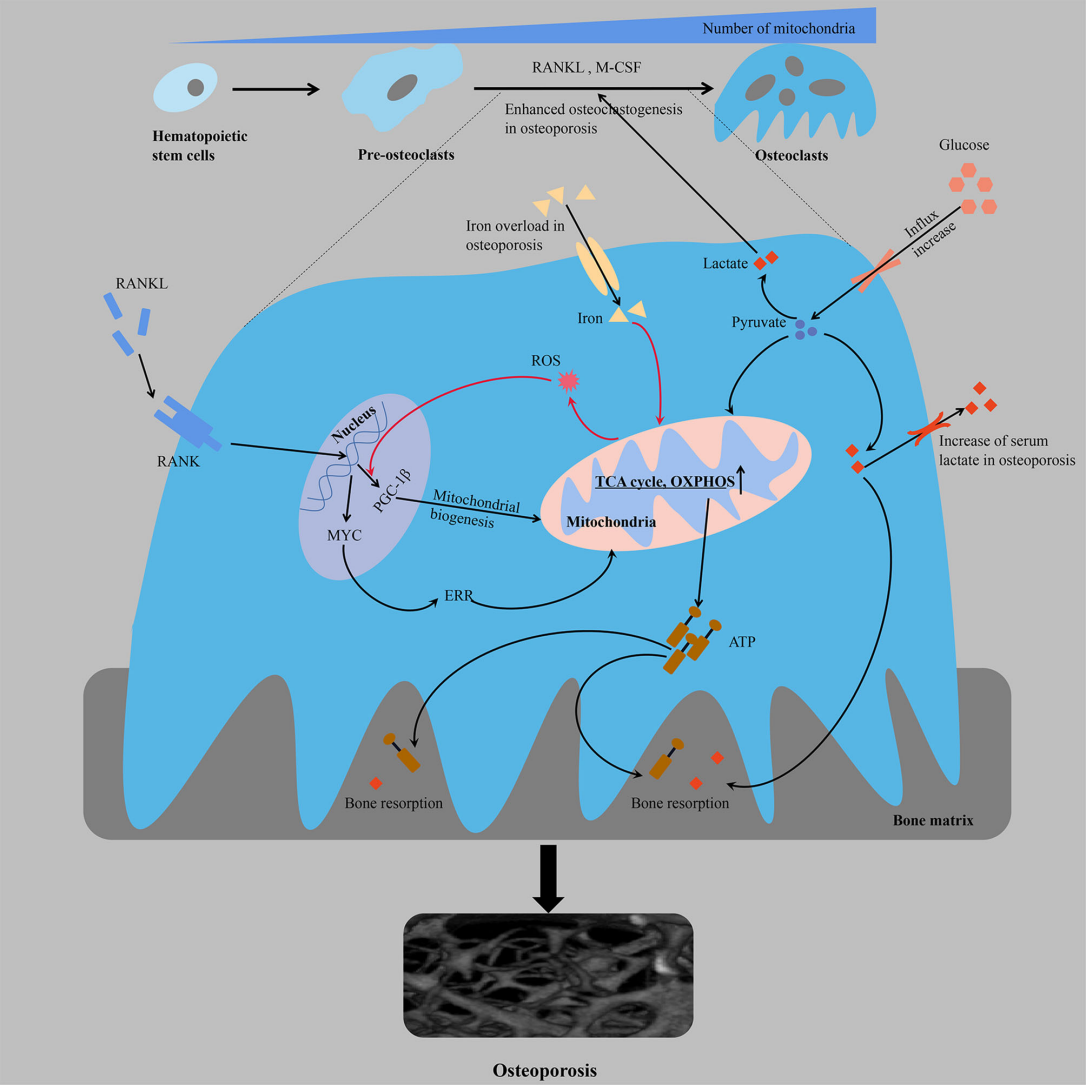
From the perspective of energy metabolism, over-differentiation of osteoclasts leads to osteoporosis. Multifactorial regulation of osteoclastogenesis is accompanied by an increased mitochondrial number and increased cellular respiration and ATP production. The orange line represents a positive feedback mechanism. ATP, adenosine triphosphate; ERR, estrogen receptor-associated receptors; M-CSF, macrophage colony stimulating factor; OXPHOS, oxidative phosphorylation; PGC-1β, peroxisome proliferator-activated receptor gamma coactivator-1β; RANK, receptor activator of NF-kB; RANKL, receptor activator of nuclear factor kappa-B ligand; ROS, reactive oxygen species; TCA cycle, tricarboxylic acid cycle.

The differentiation of monocytes into osteoclasts ensures the formation of bone-resorbing mature osteoclasts, which is a process regulated by multiple factors *in vivo* that consists of homeopathic changes in energy metabolism (**Figure 1**). There is a significant increase in glucose uptake during osteoclastogenesis [the glucose transporters GLUT1 and GLUT3 are mainly expressed in the osteoclast precursors, whereas GLUT1 is upregulated in the mature osteoclasts (38)]. Osteoclast differentiation is accompanied by an increase in the number of mitochondria (20, 39), and the enhanced oxygen consumption rate (OCR) and upregulated expression of enzymes involved in glycolysis [e.g., hexokinase, phosphofructokinase and pyruvate kinase (38, 40)], TCA cycle and OXPHOS, which indicates increased energy production (31). In addition, while osteoclast differentiation mainly depends on glucose as an energy source, the threshold is not yet clear (either too high or too low glucose levels are likely to negatively regulate bone resorption activity). The results of transcriptional and proteomic studies showed that the average expression of nucleoproteins in OCs was downregulated compared with osteoclast precursors. Moreover, the average expression of mitochondrial proteins was significantly higher in OCs (transcriptomics and proteomics were highly consistent), and the expression of proteins involved in OXPHOS, including complex I, IV and ATP synthase, were also significantly higher than the average expression levels of mitochondrial proteins in OCs. Proteins involved in catabolism pathways (including fatty acids, glycerol, glucose and amino acids) were upregulated in OCs, and complex II involved in the TCA cycle showed a greater increase in maturation than other complexes, suggesting that OCs activate the TCA cycle to increase ATP production (37). Jeong et al. found that RANKL-induced osteoclastogenesis was associated with increased activity of lactate dehydrogenase (LDH) to activate glycolysis, a process that relies on increased expression of proton-linked monocarboxylate transporters (MCT1, 2, 3, 5 and 8), excrete lactate from cells produced by increased glycolysis, reduce toxicity and enhance local acidosis to promote bone resorption, and provide positive feedback to stimulate their differentiation (41). These findings were also consistent with changes in serum metabolomic profiles in ovariectomized mice during the onset of osteoporosis, in which the serum levels of glucose and lactate were significantly increased (42). The proton pump V-ATPase consists of a peripheral catalytic V1 domain and a membrane proton channel V0 domain. The V-ATPase subunit plays an important role in the biological and physiological functions of osteoclasts, and functional abnormalities in these subunits are usually associated with osteoporosis. Partial loss of ATP6V1H function can lead to osteoporosis/osteopenia, and mutations in T-cell immune regulator 1 (TCIRG1), also known as V-ATPase V0 subunit A3, which is responsible for secreting acid, cause autosomal recessive osteopetrosis in humans (43–46). Osteoclastic differentiation, acid secretion and skeletal rearrangement are continuous physiological processes that ensure the bone resorptive capacity of mature osteoclasts. In addition, malate dehydrogenase (MDH) regulates the malate-aspartate shuttle between the cytoplasm and mitochondria relying on the coenzyme nicotinamide adenine dinucleotide (NAD^+^/NADH), thereby regulating cellular energy metabolism and interfering with osteoclast differentiation and function (47). MDH1 located in the cytoplasm catalyzes the reduction of oxaloacetate to malate, which is further converted to oxaloacetate by MDH2 in mitochondrial matrix. RANKL treatment has been shown to increase the expression of MDH1 in osteoclastogenesis, and MDH1 knockdown not only leads to decreased production of ATP but also to decreased expression of c-FOS and nuclear factor of activated T-cells 1 (NFATC1), which is a key transcription factor involved in osteoclast formation (47).

Osteoclast differentiation is associated with mitochondrial biogenesis, which may not be coupled despite pathway crossover. Compared with monocyte precursors, mature osteoclasts contain larger mitochondria with more abundant cristae and considerable structural remodeling (48, 49). The formation of mitochondrial clusters around the nucleus may be crucial for the efficient provision of biosynthetic functions, while the production of ATP in the peripheral regions of the cell may require complex networks in the basal regions, which are responsible for the depletion of bone resorption (50). Among the regulatory factors involved in mitochondrial biogenesis, the expression of peroxisome promoter-activated receptor coactivator 1 β (PGC-1β) is significantly and specifically induced during osteoclastogenesis. The mechanism is as follows: cAMP response element-binding protein (CREB) activates the downstream receptor activator of NF-kB (RANK) and immunoreceptor tyrosine-based activation motif (ITAM) in the process of osteoclastogenesis to induce transcription of PGC-1β and then stimulates mitochondrial biogenesis, resulting in an increased demand for iron, which is beneficial to the electron transport chain (ETC). Moreover, the increased intake of iron through upregulated transferrin receptor protein 1 (TFR1) induces mitochondrial respiration to ensure a sufficient energy supply, and increased production of reactive oxygen species (ROS) through an NADPH oxidase (nicotinamide adenine dinucleotide phosphate oxidase)-dependent process accelerates the transcription of PGC-1β through a positive feedback mechanism (51). Consistent with *in vivo* studies, osteopenia in ovariectomized or posterior limb dislocation mice is characterized by iron accumulation in the bone, and iron overload leads to osteoporosis by promoting osteoclast bone absorption, which is mostly attributed to its regulation of mitochondria (52). The use of iron chelating agents reduces iron levels in osteoclasts and inhibits the enzymatic activities of mitochondrial complexes I, II and III, leading to a significant decrease in the ATP content and inhibition of the mitogen-activated protein kinase (MAPK) signaling pathway to restore bone mass (53). The over-differentiation of osteoclasts during the occurrence and development of osteoporosis is complementary to the occurrence of mitochondria, which ensures a sufficient energy supply for the synthesis of proteins and factors related to differentiation. Nuclear-encoded protein NADH: ubiquinone oxidoreductase iron-sulfur protein 4 (NDUFS4) is essential for mitochondrial complex I assembly. NDUFS4 deficiency inhibits osteoclastogenesis by suppressing mitochondrial complex I activity, resulting in reduced bone resorption and increased bone mass. Moreover, in a model of inflammatory bone loss induced by lipopolysaccharide (LPS), NDUFS4 knockout restores bone mass, suggesting that mitochondrial complex I inhibition may provide a therapeutic direction based on the energy required for inflammatory osteoporosis (54). MYC is a critical upstream transcription factor in the metabolic reprogramming of oxidative phosphorylation in osteoclasts. Genetic studies have shown that osteoclast-specific MYC-deficient mice exhibit a deficiency in osteoclast development and do not exhibit ovariectomy-induced (OVX-induced) osteoporosis (55). The enhanced mitochondrial respiration (such as the TCA cycle and oxidative phosphorylation, oxygen consumption, ATP production, and respiratory capacity and reserve) of osteoclasts induced by RANKL depends on MYC/ERRα, and NFATc1-independent pathways downstream of MYC mediate the ability of RANKL/RANK signaling to influence osteoclasts (55, 56). Metabolic inhibition of OXPHOS is a feasible direction for the treatment of osteoporosis. XCT790 has been shown to effectively reduce oophorectomy OVX-induced bone loss in mice by inhibiting the metabolic reprogramming of OXPHOS during osteoclast progenitor cell differentiation through the inhibition of estrogen receptor-associated receptors (ERRs) (55). The effect of OXPHOS on osteoclast formation can be further inhibited by the respiratory chain disruptor rotenone, thereby eliminating bone loss due to estrogen deficiency in mice (57, 58).

### Proliferation and Senescence

**Figure 2:**
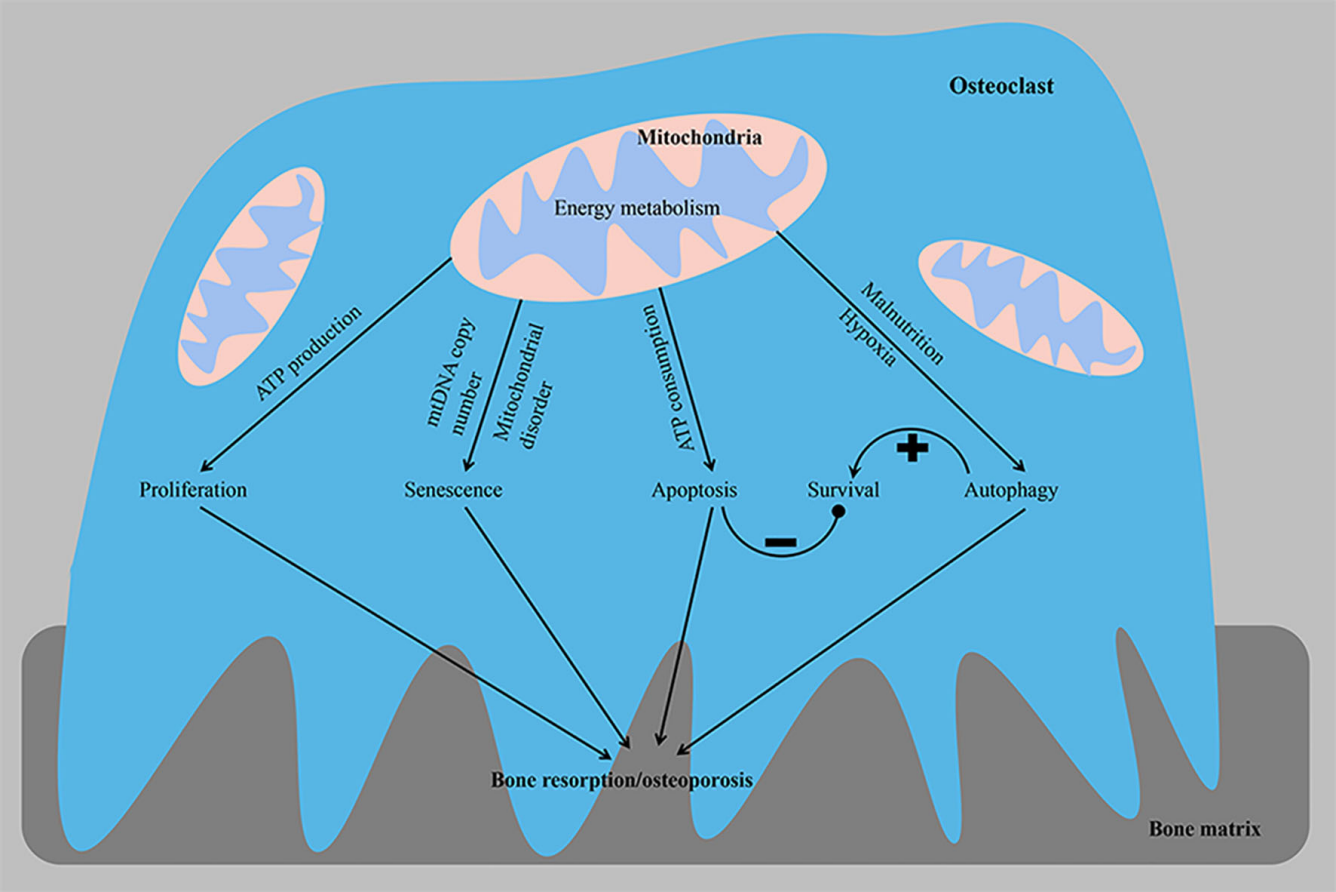
From the perspective of energy metabolism, abnormal osteoclast proliferation, senescence, apoptosis and autophagy phenotypes lead to osteoporosis. The imbalance in homeostasis between osteoclast survival and the bone resorptive capacity is a key factor contributing to the development of osteoporosis. ATP, adenosine triphosphate; mtDNA, mitochondrial DNA. –, negative control; +, positive control.

Cell proliferation is closely related to the regulation of the cell cycle process. The occurrence and development of osteoporosis are closely related to the excessive proliferation of osteoclasts (**Figure 2**). Patients with osteoporosis express the long non-coding RNA (LncRNA) colorectal neoplasia differentially expressed (CRNDE) at high levels, and this LncRNA stimulates the proliferation of osteoclasts through the PI3K/Akt signaling pathway, a process that is inhibited by estrogen (59). Furthermore, compared with precursor cells, RANKL-induced OCs exhibit reduced expression of proteins participating in the cell cycle to save energy. Transcripts encoding proteins involved in the cell cycle, cell growth and proliferation, DNA replication, recombination and repair, and free radial scanning were downregulated only in OCs compared with osteoclast precursors and intermediate osteoclasts (37). In addition, the copy number of mitochondrial DNA (mtDNA) in osteoclasts may decrease with age, which may subsequently lead to increased age-related bone resorption. Experiments on osteoclasts from 3- and 24-month-old rats showed that the copy number of mtDNA and intracellular ATP level decreased with age. A positive correlation was observed between age and osteoclast bone resorption activity (**Figure 2**). These findings from osteoclasts confirm that lower intracellular ATP levels increase bone resorption in osteoclasts, and age-related mitochondrial dysfunction, such as a reduced mtDNA copy number, may be associated with the development of osteoporosis (29).

### Apoptosis

A delicate balance exists between osteoclast formation and osteoclast survival, and mitochondria play a key role in apoptosis (**Figure 2**). Mitochondrial proteins that induce cell death [including ADP/ATP translocase (SLC25A4 and SLC25A6), HTRA serine protease2 (HTRA2), apoptosis-inducing factor 1 (AIFM1) and voltage-dependent anion channels (VDACs)] are mainly upregulated in OCs. Upregulated VDACs in mature OCs leads to increased mitochondrial permeability, which results in the release of cytochrome C and AIF from the mitochondria into the cytoplasm. As a store of pro-apoptotic molecules, mitochondria rapidly destroy osteoclasts by releasing cytochrome C, and apoptosis-promoting molecules are released to avoid bone overabsorption (37, 39). Estrogen exerts an anti-osteoporotic effect by inhibiting the activity of mitochondrial complex I in osteoclast progenitor cells to slow mitochondrial respiration and ATP generation and stimulate Bak/Bax-dependent mitochondrial apoptosis to reduce the number of osteoclasts (60). Apoptosis-sensitive phenotypes of mature osteoclasts may be partially attributed to the downregulation of ATP. The substantial ATP depletion observed following the elimination of mitochondrial transcription factor A (TFAM) accelerates the apoptosis of osteoclasts, while the higher intracellular ATP concentration induced by the upregulation of B-cell lymphoma-extra large (BCL-xL) promotes the survival of osteoclasts and exceeds the inhibitory effect of extracellular ATP, suggesting that the intracellular ATP content is a key factor regulating osteoclasts. Despite accelerated apoptosis, ATP-depleted TFAM knockout osteoclasts show increased bone resorption activity, suggesting that mitochondrial biogenesis is not a key determinant of osteoclast formation and activity (29). Mature OCs rich in mtDNA release ATP from mitochondria into the cytoplasm, and subsequent ATP depletion leads to increased bone resorption and apoptosis of OCs. This finding was also supported by the results of omics studies, in which VDAC1 and VDAC2 knockdown in osteoclast precursor cells resulted in reduced ATP production and less osteoclast formation, suggesting that VDACs were involved in cell survival through their association with energy production. These data suggest that when OCs exceed a certain size and acquire too many nuclei, they also have too many mitochondrial copies, all of which can lead to cell death (37). Overall, cellular ATP levels reflect cellular viability, including cell survival, growth and morphology, and a relatively mild ATP loss appears to regulate cell differentiation, while excessive ATP consumption induces apoptosis, which ensures strong bone resorption (47). When the ATP concentration is within the physiological range (2-5 mM), ATP is a powerful stimulator of osteoclast formation and activity. However, high concentrations of ATP inhibit osteoclast formation and activity (61).

Hypoxia also regulates the occurrence of osteoclast apoptosis, which reduces the viability of mature osteoclasts (62, 63). A hypoxic osteoclast microenvironment is formed during the development of osteoporosis. Osteoclasts reside in the resorption lacunae, where the oxygen tension is significantly lower than elsewhere in the hematopoietic chamber (64, 65). Sensitivity to hypoxia-induced cell death may result from maintaining a high ratio of oxidative phosphorylation in hypoxic environments, as evidenced by the increased expression of superoxide dismutase 2 (SOD2), a marker of mitochondrial ROS formation. A hypoxia-induced factor-1 (HIF-1) active siRNA completely reversed the chronic (48 h) hypoxic-exposure induced death of osteoclasts, with no effect on the activity of AMP-activated protein kinase (AMPK) (66). Notably, hypoxia reduced the viability of mature osteoclasts, thus suggesting that OXPHOS promotes osteoclast survival (67). The increased bone resorption mediated by osteoclasts under hypoxia is a balance between osteoclast activation and osteoclast apoptosis (63). In summary, we propose that osteoclasts engage an autonomous compensatory mechanism to avoid excessive bone resorption in the homeostasis of normal bone remodeling, and the loss of this compensatory mechanism may be responsible for the reduced apoptosis and increased differentiation of mature osteoclasts as osteoporosis develops.

The absorption of bone matrix by the ruffled border of osteoclasts leads to the release of a large amount of calcium. However, the increase in the calcium concentration in the cytoplasm is very detrimental to the survival of cells. When the homeostasis of the cytoplasmic calcium ion concentration is disturbed, cells generally undergo necrosis or apoptosis (68). The cytoplasmic calcium-binding protein calmodulin is concentrated in the cytoplasm of OCs near the ruffled border and controls intracellular calcium concentrations and the activity of ATPase, a proton pump that transfers protons produced by carbonic anhydrase II (CAII) into intracellular vesicles. As the OC absorptive activity increases, the level of cytoplasmic calcium increases, which inactivates OCs and leads to cell separation from the bone matrix, ultimately leading to the loss of a ruffled border (69–71). Osteoclasts collect Ca^2+^ by forming mitochondrial granules to reduce the concentration of Ca^2+^ in the cytoplasm. In addition, mitochondria in the absorption region of the ruffled border are rich in mitochondrial granules with a high content of Ca^2+^, while few mitochondrial granules are located in the basolateral region. Meanwhile, Ca^2+^ is also released outside of the cell *via* vesicular transport. The formation of mitochondrial granules depends on the direct transport of Na-Ca exchangers and the transport mediated by the endoplasmic reticulum, and these processes depend on the energy supplied by the respiratory chain (72). Notably, this mechanism is similar to positive feedback, and studies aiming to explore whether this autonomous mechanism of osteoclasts exists in the homeostasis of bone remodeling or when osteoclasts undergo excessive bone resorption are necessary and may provide a direction for osteoporosis therapy.

### Autophagy

Lysosomes degrade and digest damaged and denatured organelles, proteins, lipids, nucleic acids and other biological macromolecules in cells, providing raw materials for cell regeneration and repair and recycling intracellular substances. Autophagy is a dynamic process that is the key and fine-tuning process for maintaining energy homeostasis (73). Autophagy also functions in lipid clearance (lipophagy) and plays an important role in lipid metabolism, lipoprotein assembly and lipid metabolism homeostasis (74). Low levels of autophagy have been observed in all cell types under basal conditions. However, the level of autophagy may be upregulated by malnutrition or hypoxia. Autophagy activation in osteoclasts is the cause of rapid bone loss in ovariectomized mice. Selective deletion of autophagy-related protein 7 (ATG7) in monocytes reduces osteoclast differentiation and bone loss induced by glucocorticoids and oophorectomy, and the deletion of ATG5 in osteoclasts also reduces bone loss caused by estrogen deficiency (75, 76). The essential autophagy proteins ATG5, ATG7, microtubule-associated protein 1A/1B-light chain 3 (LC3) and ATG4B are involved in the polarization of osteoclasts and are closely related to the formation of osteoclast ruffled borders, H^+^ secretion from lysosomes and protein hydrolysis (75). Lysosomes accumulate acid-degrading enzymes that are transported to the bone resorption region and constitute the main components of the bone resorption lacunae (77, 78). Transcription factor EB (TFEB) overexpression results in a significant increase in the total number of lysosomes, suggesting that TFEB is a major regulator of lysosomal biogenesis. Furthermore, cells monitor lysosomal function and adapt to the degradation requirements and/or environmental signals by modulating TFEB activity. Activation of TFEB in osteoclasts is independent of nutrient levels but depends on RANKL-mediated signaling. The upregulation of specific lysosomal genes by TFEB is essential for bone matrix absorption. In mice with an osteoclast-specific TFEB deficiency, the decrease in lysosomes was accompanied by a decrease in lysosomal gene expression and a decrease in the bone absorptive capacity (79, 80). Hypoxia also plays a major role in the regulation of mitochondrial autophagy and the subsequent release of reactive oxygen species (ROS) during osteoclast differentiation and functional activation (81). In addition, activated AMPK promotes osteoclast autophagy and increases osteoclast differentiation and bone resorption by inactivating mammalian target of rapamycin (mTOR) under low-energy conditions, such as glucose or amino acid deficiency (82, 83). Autophagy inhibits apoptosis while promoting the self-renewal of intracellular substances. Autophagy plays a dual role in regulating osteoclasts. Even if autophagy is important for bone resorption, autophagy activation does not necessarily stimulate osteoclast activity (84). OPG activates the AMPK protein, resulting in the inactivation of mammalian target of rapamycin complex 1 (mTORC1). This inhibitory effect enhances autophagy, which increases the bone resorption capacity by regulating actin and the microtubule network (75, 85); however, the inhibition of mTOR downregulates the expression of digestive enzymes and induces osteoclast apoptosis (86). Autophagy also regulates the survival, differentiation and function of osteoclasts (**Figure 2**). A disorder in the energy metabolism of osteoclasts leads to disruptions in autophagy activity and the balance between bone formation and bone resorption and mediates the occurrence and progression of a variety of bone diseases, including osteoporosis (87, 88).

## Effects of Hypoxia and Oxidative Stress on Energy Metabolism in Osteoclasts

Bone metabolism homeostasis is based on the dynamic processes of osteogenesis and osteoclastogenesis. From the perspective of osteoclasts, the differentiation of monocytes into mature osteoclasts and the death of mature osteoclasts after bone resorption is a continuous process, and energy metabolism changes in different stages (glycolysis and respiration are constantly transformed in a delicate balance to support the different energy requirements in the sequential differentiation phase). The energy required for osteoclast differentiation is mainly derived from mitochondrial oxidative metabolism (increased expression of OXPHOS enzymes, complexes I, II, III and V, electron transport chain subunits, intracellular ATP levels and a higher oxygen consumption rate) (89), while the activity of peripheral cells associated with bone matrix degradation is supported by glycolysis (50). Metabolic compartmentalization is an interesting theory that aims to illustrate the energy metabolic activity of different regions of the cell in response to different biological functions of osteoclasts (e.g., bone resorption, cytoskeletal reorganization, vesicular transport, and protein synthesis). Typically, enzymes associated with the glycolysis pathway [such as pyruvate kinase M2 (PKM2) and glyceraldehyde 3-phosphate dehydrogenase (GAPDH)] are located near the actin ring of osteoclasts, where energy-producing activities associated with bone degradation occur (50). Furthermore, the amount of ATP required to pump protons into the absorption region may exceed that produced by cellular mitochondrial biogenesis, and thus, glycolysis in the cytoplasm may be a more readily available source of ATP than oxidative phosphorylation in the mitochondria and transport by ADP/ATP transporters (90). Various other metabolic byproducts also regulate bone resorption, such as pyruvate (which promotes osteoclast differentiation) and H^+^ ions (which make the extracellular environment acidic). Acidosis is known to play a direct role in osteoclast function by upregulating genes responsible for osteoclast adhesion, migration, survival, and bone matrix degradation and increasing reactive oxygen species production to stimulate osteoclast formation and absorptive activity (70, 91–93).

Osteoclasts are hypoxia-responsive cells. In vivo studies have found that an increase in HIF-1 levels activates osteoclasts during osteoporosis (94). In vitro, HIF-1 expression is upregulated in osteoclasts stimulated with hypoxia, and glycolytic enzymes driven by HIF-1, such as lactate dehydrogenase A (LDHA), glucokinase (GCK), PKM2 and phosphofructokinase E1 (PFK1), are also upregulated. HIF-1 interference not only inhibits hypoxia-mediated glycolysis (including a decrease in GLUT1 expression) but also inhibits hypoxia-induced acid secretion and bone resorption. In addition, inhibitors of glycolytic enzymes block acid secretion from hypoxic osteoclasts, and the chemical inhibition of PFK1 and LDHA significantly reduces hypoxia-induced osteoclast formation, but the inhibition of GCK and PKM2 does not produce significant changes (95). Morten et al. also found that hypoxic osteoclasts (24 h, 2% O_2_) increased ATP production by 56% through increased oxygen consumption and HIF-1α-dependent glucose uptake mediated by GLUT1 and ETC activity. When cultured on dentine with an osteoclast resorption substrate, the increase in intracellular ATP levels induced by hypoxia was not significant, suggesting that ATP is a potentially useful treatment for bone resorption. Even in a hypoxic environment, switching to anaerobic glycolysis alone does not support the increased requirement for ATP for bone resorption, and mitochondrial flux plays a greater role. The accumulated ROS exert positive feedback (promoting osteoclastic differentiation and enhancing bone resorption), which may be an adaptive mechanism to achieve rapid bone resorption in a short time (66). HIF-1α knockdown does not affect osteoclast differentiation but inhibits bone resorption by mature osteoclasts. This change in activity is accompanied by a reduction in the expression of glycolysis-related enzymes and proteins, while typical osteoclast marker genes, such as the transcription factor NFATC1 or protease CTSK, were not affected (38, 96). In addition, hypoxia inhibits proline hydroxylation, allowing HIF-1 to stably coordinate and construct transcriptional complexes in cells overexpressing HIF-1. Hypoxia enhances the activity of the E2F1-dependent glycolysis pathway by inactivating copper metabolism domain containing 1 (COMMD1), an upstream negative regulator of osteoclastogenesis under normoxic conditions, thus promoting the formation of osteoclasts (97). In vitro studies have shown that fatty acid oxidation is thought to drive ATP production in osteoclasts (98). However, hypoxic osteoclasts accumulate neutral lipids, suggesting that hypoxia inhibits the input and/or utilization of mitochondrial fatty acids. Glutamine is decomposed into α-ketoglutarate, which further participates in the mitochondrial TCA cycle. Hypoxic osteoclasts increase glutamine uptake by 4.1-fold through a mechanism depending on HIF-2α. However, in the presence of a low oxygen concentration, glutamine withdrawal has no effects on ATP production and osteoclast survival, whereas the removal of glucose significantly inhibits both processes, suggesting that glucose is the primary substrate for ATP production and that glutamine may be used in biosynthesis (nucleotides and hexose amine) (66). Reduced intracellular ratios of ATP : ADP or ATP: AMP activate AMPK and inhibit osteoclast differentiation and function (99). In osteoclasts, hypoxia inhibits AMPK phosphorylation and inactivates AMPK, which may be due to an increased intracellular ATP : AMP ratio, and blocks the mechanism by which hypoxia induces AMPK activation by facilitating dephosphorylation, which is necessary for hypoxic reabsorption to occur (66). Osteoblasts release a large amount of ATP through vesicle-mediated exocytosis, resulting in a significant increase in the extracellular concentration of adenosine during hypoxia or inflammation (100, 101). In addition, hypoxic osteoclasts also secrete ATP to increase the concentration of adenosine. In hypoxic osteoclasts cultured *in vitro*, the concentration of extracellular adenosine (the substrate of adenosine A_2B_ receptor) is substantially increased, which is able to activate the A_2B_ receptor without any other stimulus. Activation of A_2B_ receptor signaling further regulates cytoskeletal rearrangement to increase bone resorption (including the interaction between A_2B_ receptor and actin cytoskeleton after stimulation through recruitment to the plasma membrane and initiating the interaction with actin-related ezrin and actin-1) (102–104). Hypoxia-induced accumulation of extracellular adenosine activates the A_2B_ receptor to further activate HIF-1α signaling, increasing glucose uptake and glycolysis to provide energy for enhanced bone resorption, which represents a positive feedback regulatory mechanism (105). However, some studies suggest that extracellular ATP may alter the morphology, survival and bone resorption activity of mature osteoclasts. A physiological concentration of extracellular ATP inhibits osteoclastic bone resorption through the purinergic receptor (P2RX7) due to a disrupted cytoskeletal structure that depends on the mislocalization of protein tyrosine kinase (PYK2), which displays a diffuse actin distribution, resulting in cellular autonomic defects in bone resorption. Apyrase treatment or P2RX7 ablation reverses this inhibition (29, 106).

In general, cellular adaptation to hypoxia usually requires switching to anaerobic metabolism, reducing the production of ATP to prevent the accumulation of reactive oxygen species (107). This survival mechanism is mainly mediated by hypoxia-inducible transcription factors (HIF-1 and HIF-2). In addition, HIF signaling inhibits the conversion of pyruvate dehydrogenase (PDH) to acetyl-CoA by increasing the expression of PDH kinase (PDK) in response to hypoxia, which reduces TCA flux and ROS accumulation. At the same time, HIF induces the expression of BCL-2 interacting protein 3 (BNIP3) to compete with beclin-1 for binding to BCL-2, resulting in the release of beclin-1 to stimulate mitochondrial autophagy and a decrease in ROS accumulation (108). However, mitochondrial ROS accumulate in hypoxic osteoclasts, as PDH is not inhibited and BNIP3 expression does not increase at low oxygen levels. The increased ROS are mainly produced by mitochondrial activities, especially complexes I and II of the ETC, and the reverse electron transfer between these complexes and molecular oxygen. Alternatively, ROS are also produced by the activation of NADPH oxidase (NOX2) in the cytosol (109). Macrophage colony-stimulating factor induces precursor cells to differentiate into osteoclasts and increases intracellular ROS levels in a NOX2-dependent manner. In vitro, the NOX2 mRNA is highly abundant in the mouse macrophage line RAW264.7, and a RANKL treatment significantly reduces the expression of the NOX2 mRNA and increases osteoclastogenesis. Notably, NOX2-dependent acute ROS formation supports osteoblast formation, while persistent NOX4-dependent H_2_O_2_ formation supports osteoclast formation in bone resorption (110). Rac1, part of the NOX complex (110), tightly regulates the actin cytoskeleton and is therefore a key factor involved in the production of reactive oxygen species. Rac1/Rac2-deficient mice show an osteopetrotic phenotype despite a normal number of osteoclasts, indicating that the Rac protein is more important for bone resorption than differentiation in the body (111). Mitochondrial ROS may be more important in mature cells than in differentiated cells. Therefore, simple supplementation with antioxidants may prevent osteoclast-mediated bone loss on the one hand and promote bone formation by osteoclasts on the other hand. In particular, in the case of noninflammatory osteoporosis, targeted interference with individual NADPH oxidase activity will help patients simply eliminate all ROS (110). Intracytoplasmic ROS inhibit glycolytic enzymes (such as GAPDH and PKM2), leading to the accumulation of glycolytic intermediates, and ROS increase metabolic flux through the pentose phosphate pathway. ROS also inhibit energy metabolism in mitochondria, including the TCA cycle and β-oxidation of fatty acids. Regardless of the source of ROS, studies have shown that excessively elevated ROS levels weaken the differentiation and function of osteoclasts and help to limit bone resorption (70, 88, 112). Therefore, cytoprotective mechanisms are needed to maintain a safe balance between osteoclast formation and osteoclast damage. Cytoprotective mechanisms include the induction of scavenging enzymes such as superoxide dismutase, catalase or glutathione, which ultimately reduce reactive oxygen species, but the levels of these proteins are thought to be attenuated during differentiation. Osteoclasts are equipped with sensor proteins, and once a critical threshold is reached, the integrity of osteoclasts may be compromised, thus activating cellular defense mechanisms. Thioredoxin will be released as an antioxidant under oxidative stress conditions (113). The expression of thioredoxin-1 increases during osteoclast differentiation, which enhances the binding of the transcription factors AP-1, NF-kB and NFATc1 to their DNA recognition sites to support osteoclast differentiation (114, 115). In conclusion, oxidative stress exerts a bimodal effect on osteoclasts: moderate stress promotes osteoclast formation, while severe stress inhibits osteoclast absorption and limits the lifetime of osteoclasts (116, 117).

## Other Substrates for Energy Metabolism

### Amino Acids

Amino acid metabolism also plays a key role in regulating the occurrence of osteoclasts, but few studies have examined this process. In addition to its direct contribution to protein synthesis, glutamine is also an important energy source and an essential carbon and nitrogen donor for the synthesis of amino acids, nucleotides, glutathione and aminohexose. Glutamine is decomposed to form α-ketoglutarate, which enters the tricarboxylic acid cycle and is converted to citrate through oxidation (118, 119). L-glutamine is another substance that plays a role in osteoclast formation. The concentration of glutamine in culture medium influences the formation of osteoclasts, and the consumption of L-glutamine inhibits the differentiation and function of osteoclasts. The expression of Na^+^-dependent glutamine neutral amino acid transporter B (0) increases during differentiation and plays an important role in later differentiation stages (39, 120). In addition, Morten and Zhou et al. reported that hypoxia stimulates glutamine consumption by osteoclasts (66, 118). Inhibition of MYC eliminates osteoclast differentiation and function and inhibits the expression of the amino acid transporter ASCT2 (SLC1A5) and glutaminase. Based on the coordination of HIF-1a and MYC, the uptake of glucose and glutamine and the utilization of carbon sources are essential for the development of osteoclasts and bone resorption (38). However, an increase in glutamine intake may mainly promote biosynthesis because the intake of glutamine has no effect on ATP produced by any pathway (118). In addition, recent studies have found that RANKL-induced osteoclastogenesis mainly depends on the presence of extracellular arginine, and 43 RANKL-induced proteins are antagonized by recombinant arginase 1 (RecArg1), which metabolizes arginine to urea and ornithine treatment, including proteins related to osteoclast formation and the actin cytoskeleton, DNA replication and cell cycle. For energy, TCA oxidative metabolism is an important metabolic link through which arginine participates in interference. Studies using a ^13^C-labeled tracer found that the arginine involved in the urea cycle is metabolized to citrulline and then forms argininosuccinate, which is converted into fumarate in the TCA cycle and participates in supplying energy. Extracellular arginine is further depleted by RecArg1 through conversion to ornithine and urea, which reverses the accumulation of malic acid, fumaric acid, succinic acid and α-ketoglutarate induced by RANKL, diminishes the expression of enzymes involved in the TCA cycle, and upregulates the expression of enzymes participating in serine and purine biosynthesis (phosphoenolpyruvate carboxykinase2, enolase2 and phosphoglycerate dehydrogenase). In addition, the excessive consumption of arginine restores osteoclastogenesis by supplementing arginine-succinic acid and citrulline, but direct supplementation of TCA intermediate products such as α-ketoglutarate does not exert a rescue effect. In addition, the essential amino acids required for osteoclastogenesis, including α-ketoisocaproate, ketoisoleucine and phenylpyruvate, are the intermediate metabolites of leucine, isoleucine and phenylalanine, respectively, and those metabolites regulate the ATP supply through acetyl-CoA entry into the TCA cycle (121). α-Ketoisocaproate, ketoisoleucine and phenylpyruvate also restore the inhibitory effect of the lack of parent amino acids on osteoclastogenesis (122). The effect of arginine deprivation on osteoclast formation does not depend on mTORC1 activity or overall transcription and translation inhibition. Intracellular proteins involved in lysine degradation are enriched during osteoclasts, and the biosynthesis of tyrosine, phenylalanine, and tryptophan is activated (37, 123). In summary, amino acids (and their metabolites) in the body may directly participate in energy metabolism pathways (including glycolysis and the TCA cycle) and may also be linked to amino acid-dependent transcription of TCA cycle enzymes and related metabolite production.

### Fatty Acids

Fatty acids are closely related to bone metabolism. The existing research mainly focuses on the regulation of osteoclast phenotypes (such as differentiation, apoptosis, proliferation, activation, and skeletal rearrangement) by various fatty acids, and many scholars have also summarized relevant studies. These studies are mostly based on the role of fatty acids as exogenous ligands to regulate intracellular transcription and translation pathways [c-Jun N-terminal kinases (JNK), extracellular signal-regulated kinases (ERK) and protein kinase B (PKB)], which may also affect energy metabolism as the signal cascade is amplified (124–126). However, our purpose is to summarize the role of fatty acids in the occurrence and development of osteoporosis, in which fatty acids directly regulate cellular energy metabolism (glycolysis, oxidative phosphorylation, etc.) From the perspective of substrates, exogenous fatty acids affect osteoclast energy metabolism to interfere with the phenotype and functional activation of osteoclasts. Fatty acids in osteoclasts are obtained from nascent adipogenesis or passive diffusion after the lipolysis of adipocytes and triacylglycerol. Fatty acids enter the mitochondrial matrix and are used as substrates for fatty acid oxidation (FAO), which maintains the energy balance. Dodds et al. found that osteoclast formation is related to enhanced fatty acid oxidation (98). A plasma metabolomics study involving 34 adult women found that the serum C-terminal telopeptide (CTX) concentration, an index of bone resorption, was related to lipid and fatty acid metabolism pathways, but not carbohydrate metabolism. Osteoclasts in the active bone resorption state show a high capacity for fatty acid β oxidation, suggesting that the high energy state of active osteoclasts may be mainly supported by lipid catabolism (127). Short-chain fatty acids (SCFAs) inhibit osteoclast production *in vivo* and *in vitro* (128). Propionate (C3) and butyrate (C4) cause metabolic reprogramming (conversion of oxidative phosphorylation to glycolysis) in osteoclasts and downregulate TNF receptor associated factor 6 (TRAF6) and NFATc1, leading to a decrease in osteoclastogenesis that may depend on oxidative stress and acid accumulation, and treatment with C3 and C4 effectively prevent OVX-induced bone loss (129). In addition, some studies have found that osteoclasts cultured in low serum [1% fetal bovine serum (FBS)] exhibit a significant increase in the expression of proteins related to fatty acid oxidation, suggesting that fatty acid oxidation is involved in the formation of osteoclasts and the supply of ATP required for bone resorption (130). In the mitochondria of osteoclasts cultured with low serum concentrations, fatty acid biosynthesis (mainly saturated fatty acids) is increased, but the underlying and molecular mechanism requires further exploration (130). Interestingly, bone resorption experiments using osteoclasts attached to bone slices *in vitro* found that osteoclasts showed a high capacity for fatty acid β-oxidation, although the lack of glycolytic-related enzymes in such osteoclasts has also been observed (98).

## The Coupling of Systemic Energy Metabolism Disorder With Osteoclast Energy Metabolism

Bone is a dynamic metabolic tissue. Osteoclasts are sensitive to environmental changes, such as inflammation, diet, exercise and other metabolic diseases, or mutations in key metabolic proteins and metabolic pathway disorders, resulting in the metabolic adaptation of osteoclasts. In the process of cell differentiation or activation, the energy metabolism of the cell is adjusted according to the actual needs of the cell, and the disorder of overall metabolic activity affects the relevant phenotypic changes in each cell of the body. Therefore, diseases such as hyperthyroidism, diabetes, obesity, and anorexia that cause metabolic disorders in the body are often accompanied by complications of osteoporosis, which are also based on the conversion of osteoclast energy metabolism (65, 91).

### Hyperthyroidism

Hyperthyroidism can lead to a high bone turnover rate, increase the initial frequency of bone remodeling and increase the rate of bone resorption and formation. The imbalance between bone resorption and bone formation shortens the bone remodeling cycle, resulting in a net loss of bone in each remodeling cycle, which accelerates the occurrence and development of osteoporosis (131, 132). Studies have identified 23 enzymes involved in glycolysis, 20 enzymes involved in the TCA cycle and 43 enzymes involved in oxidative phosphorylation that are upregulated in the bones of hyperthyroid rats, indicating a significant increase in ATP synthesis and glucose catabolism in the femur tissues of hyperthyroid rats. Thyroid hormones stimulate various metabolic enzymes involved in the metabolism of glucose, lipids and proteins. Excessive thyroid hormone levels may promote osteoclastogenesis and enhance absorptivity by stimulating the glycolysis, TCA cycle, and oxidative phosphorylation pathways (133). Therefore, inhibition of the overactivation of the glycolysis, TCA cycle, and oxidative phosphorylation pathways and normalization of energy metabolism may provide therapeutic targets for hyperthyroidism-related osteoporosis.

### Diabetes

The relationship between diabetes [both type 1 diabetes (T1D) and type 2 diabetes (T2D)] and osteoporosis is complicated. Patients with diabetes have lower bone turnover, a lower bone density, and increased fracture risk (134). The mechanism of bone damage in patients with diabetes is not yet clear. Diabetes is a metabolic disease characterized by chronic hyperglycemia and metabolic disorders. One possible mechanism is that the properties of collagen (the most abundant bone protein) are impaired due to complications such as hyperglycemia, oxidative stress, and accumulation of advanced glycation end products (135). Hyperglycemia can cause the dysfunction of many metabolic systems, including bones, indicating that energy metabolism in turn affects cell differentiation. Glucagon-like peptide 2 (GLP2), a molecule regulating energy metabolism, is a beneficial factor in glucose metabolism in mice with high-fat diet-induced obesity. It prevents osteoporosis by inhibiting osteoclast proliferation and promoting apoptosis (136). Osteoclasts cultured on the bone slices transport glucose almost twice as fast as the osteoclasts away from bone to ensure a sufficient energy supply for bone resorption. At the same time, the acidic microenvironment of the bone resorption cavity decarboxylates osteocalcin derived from osteoblasts and activates its function, which promotes the expression of insulin in pancreatic β cells and reduces blood sugar levels (137–139). Bone resorption in physiological bone remodeling homeostasis is beneficial for controlling blood glucose levels in the body, and overactivated bone resorption in response to hyperglycemia may lead to the weakening or loss of this compensatory adaptation mechanism. Intracellular energy production is actively adjusted according to changing needs and is determined by the level of energy consumption, not by the extracellular concentration of metabolic substrates. Therefore, at any basic glucose and pyruvate level, the rates of glycolysis and oxidative phosphorylation are adapted to the energy needs of precursors for proliferation, growth and fusion, as well as osteoclast differentiation, fusion and growth. A conceivable hypothesis is that the gradual increase in energy consumption during differentiation provides a tolerance space for the increase in energy production, and an excessive amount of energy substrates, such as glucose, pyruvate, and citrate, promotes the recognition of differentiation signals (91). In a mouse model of type 2 diabetes, moderate hyperglycemia (approximately 10 mM serum glucose) is associated with increased osteoclast formation (140, 141). In contrast, hyperglycemia (40 mM) inhibits RANKL-induced osteoclast formation (31, 142), which can be explained by metabolic effects such as a lower oxygen consumption rate at higher glucose levels (143). In addition, the optimal glucose level for osteoclast development and its relationship with local oxygen tension in the body remain to be determined (38). The enhanced OC activity in diabetic animal models may not be a direct consequence of hyperglycemia. Studies have found that bone marrow stromal cells (BMSCs) of mice with type 2 diabetes oversecrete succinate (an intermediate metabolite in the TCA cycle that participates in regulating the energy supply) that binds to succinate receptor 1 (SUCNR1) of osteoclasts as an extracellular ligand and stimulates osteoclastogenesis *in vitro* and *in vivo* without increasing the number of OC precursor cells. In addition, succinate directly stabilizes HIF-1α signaling to stimulate osteoclastogenesis. However, an interesting finding is that the intracellular accumulation of succinate induced by inhibiting succinate dehydrogenase (SDH) suppresses osteoclast formation (144). Glutamate levels are significantly increased in diabetic mice and humans, which may also exert osteoclastic resorption effects from the perspective of ligands and energy substrates (144, 145). The roles of products of glycolysis and the TCA cycle as intracellular metabolites are speculated to be different from their roles as extracellular molecules.

### Obesity

Hyperlipidemia increases bone resorption (146). Patients with a low bone mineral density and osteoporosis have higher lipid levels, and elevated serum levels of low/very low density lipoprotein (LDL/VLDL), glucose, lactate, and lipids have been detected in ovariectomized mice (42). Lipids and vLDL/LDL are involved in lipid metabolism and produce fatty acids, glycine and cholesterol. The increases in the levels of fat and free fatty acids induced by OVX promote the formation of osteoclasts, leading to bone loss (147, 148). Studies have found that the integrity of osteoclast lipid rafts interferes with the activity of V-ATPase, and the disruption of rafts with cholesterol-sequestering agents will impair the function of V-ATPase, thereby inhibiting bone resorption activity (149). Hyperlipidemia and bone marrow fat are associated with a decreased bone density in the body, suggesting that lipids contribute to bone loss (150). Free fatty acid and fat levels in the bone marrow of obese mice are significantly increased (151). Dietary fatty acids (FAs) are an important source of energy, and diets containing excessive amounts of saturated fatty acids (SFAs) or a high ratio of pro-inflammatory omega-6 polyunsaturated FAs (PUFAs) to anti-inflammatory omega-3 PUFAs may lead to foodborne obesity. FAs affect intracellular receptors, such as peroxisome proliferation-activated receptors (PPARs are composed of PPARα, PPARδ and PPARγ), and then regulate fatty acid oxidation, fat formation, energy balance and lipid biosynthesis. The activation of PPARα and PPARδ inhibits osteoclastogenesis and bone resorption (152, 153), while PPARγ positively regulates osteoclastogenesis (154). Long-term use of rosiglitazone leads to bone loss and a reduction in bone mass. In monocytes, rosiglitazone activates PPARγ to increase the expression of the downstream molecules ERRα and PGC-1β, and it activates the ERRα pathway through a ligand-dependent mechanism. Finally, the two processes jointly lead to mitochondrial biogenesis (including target genes involved in the TCA cycle and OXPHOS), resulting in osteoclast differentiation and bone loss (155, 156). Our review found that the existing studies of obesity and osteoporosis mostly focus on obesity and the phenotypic regulation of osteoblasts and osteoclasts, and most of these changes are explained by adipokines, including leptin and adiponectin, signaling through paracrine and endocrine mechanisms (157, 158). However, few studies have focused on circulatory abnormalities in lipids and fatty acids interfering with the energy metabolism of osteoclasts by altering substrates, ligands or components of specific cellular functional complexes in obese individuals.

### Anorexia

A decreased bone mineral density (BMD) is one of the main complications of anorexia nervosa, and the body is in a state of hunger and low energy, leading to a large number of metabolic and physiological changes (159). Anorexia nervosa is associated with significant weight loss, a low BMD and increased fracture risk (160). The BMD of the spine in normal weight women with bulimia nervosa is significantly lower than that in healthy women. Based on these data, the adverse effects of dietary disorders on BMD are not attributed solely to weight loss (161). Many factors, including hormones, endocrine factors and nutrition, are involved in the mechanism of bone loss in these patients. Low serum (1% FBS) culture increases the activity of osteoclasts (tartrate resistant acid phosphatase (TRAP), CTSK, and MMP-9 expression are upregulated), and the biological functions of other upregulated proteins are mainly enriched in lipid homeostasis, ATP hydrolysis coupled proton transport, fatty acid beta-oxidation using acyl-CoA dehydrogenase, translation, TCA cycle (also termed citrate cycle) and mitochondrial translation, indicating that mitochondrial activity and energy metabolism are significantly increased during the formation of osteoclasts in a low serum culture system (130). These findings may also reveal the pathogenesis of osteoporosis secondary to anorexia and why bone resorption is also extensive when energy substrates are insufficient.

## Epigenetic Regulation of Osteoclast Energy Metabolism During the Development of Osteoporosis

Metabolic pathways control gene expression through epigenetic modification because most of the substrates and cofactors necessary for epigenetic modification can be produced through bioenergetic pathways. At the same time, epigenetic factors also regulate enzymes or substrates of energy metabolism and then regulate energy conversion, eventually leading to alterations in the cell phenotype. Epigenetic changes may be a potential explanation for the association between the *in vivo* characteristics and *in vitro* OC formation/activity of osteoporosis. Reversible modifications of DNA, such as histone acetylation, methylation, phosphorylation, and ubiquitination, alter transcriptional mechanisms and regulate gene expression and osteoclastic differentiation and activity (162). We only identified very few studies on the epigenetic regulation of osteoclast energy metabolism during the occurrence and development of osteoporosis. This topic requires further examination in the future.

### Acetylation

Acetyl-CoA produced by mitochondria in the TCA cycle is a key cofactor and substrate for the acetylation reaction. It is used as an acetyl donor by histone acetyltransferase and is known to exist in two separate banks: one in the mitochondria and the other in the cytoplasm (90). Changes in the cellular metabolic pathway and substrate flux not only control ATP production but also regulate gene expression. At the same time, the availability of substrates and ATP demand of cells also regulate the conversion of energy metabolism. Sirtuin 3 (SIRT3) is a mitochondrial (NAD)+-dependent deacetylase that has been identified as a negative regulator of osteoclast formation (163, 164). SIRT3 inhibits RANKL-mediated osteoclast formation by stabilizing the AMPK protein, and the production of osteoclasts in SIRT3-deficient mice is increased (164). RANKL stimulates ROS production, which promote osteoclastogenesis (165); however, RANKL also increases the expression and activity of SOD2 and SIRT3, which activates SOD2 by deacetylating lysine 68 to exert negative effects on osteoclast formation and prevent excessive OC formation in physiological bone metabolism (163). In addition, the activity of PGC-1 is finely regulated by pathways that detect the metabolic state of cells. In particular, sirtuin 1 (SIRT1) and AMPK have been shown to regulate the activity of PGC-1 through deacetylation and phosphorylation, respectively, forming a coordinated mitochondrial metabolic regulatory network (166, 167). ROS are essential for the formation and functional activation of osteoclasts during the occurrence and development of osteoporosis. Oxidative stress will promote the interaction of SIRT1 and FoxO, thereby promoting the deacetylation of FoxO, which may activate FoxO and further initiate subsequent transcriptional regulation. The targeted deletion of SIRT1 in osteoclasts results in low bone mass (168), indicating increased osteoclastogenesis or bone resorption or osteogenic inhibition. Therefore, a reasonable hypothesis is that the mechanism by which SIRT1 inactivation inhibits the deacetylation of FoxO and promotes osteoclastogenesis and bone resorption may exist during the development of osteoporosis (169). However, this mechanism differs from the idea of increased oxidative stress, and thus the ROS-Sirtuin axis may be a compensatory regulatory mechanism when physiological osteoclastogenesis occurs.

### Methylation

DNA methyltransferase 3a (DNMT3a) plays a key role in the pathological activation of osteoclast formation (170). S-adenosylmethionine (SAM) is the methyl donor of DNMT3a in the methylation reaction. Methionine adenosyltransferase (MAT) produces SAM from methionine and ATP. Both osteoclast precursor cells and osteoclasts express MAT2a. The formation of osteoclasts induced by RANKL depends on increased activity of the TCA cycle and the high ATP levels produced by oxidative phosphorylation, which participate in increasing SAM production, thereby ensuring the inhibition of interferon regulator factor 8 (IRF8, a negative regulator of osteoclastogenesis) methylation by DNMT3a. In addition, treatment with the respiratory chain inhibitors antimycin A and oligomycin eliminate this methylation inhibition. The inhibition of DNA methylation by theaflavin-3,3’-digallate reverses bone loss in osteoporosis models. Notably, RANKL stimulation does not change the level of acetyl-CoA, which is the acetyl donor for the acetyltransferase reaction (170).

## Unresolved Issues

- The changes in mitochondrial dynamics of osteoclasts during the development of osteoporosis and the amount of ATP produced by different metabolic pathways will be crucial to obtaining a complete bioenergy map. Research on the extent to which fatty acids and amino acids serve as energy substrates involved in oxidative phosphorylation during the formation of osteoclasts is lacking.
- Effects of the metabolism of hypoxic substances on the biological behavior of osteoclasts remain unclear. Therefore, the precise molecular events of osteoclast glycolysis require further study, especially under hypoxic conditions.
- Can we precisely manipulate mitochondria in a cell-type- and space-time-specific manner? Current pharmacological treatments have limitations in precisely regulating mitochondrial function.
- Research on the mechanism of secondary osteoporosis based on the regulation of osteoclast energy metabolism is still seriously insufficient. A more detailed study of bioenergy pathways at the molecular level may reveal new mechanisms of pathological bone loss and then identify new therapeutic targets.

## Summary and Hypothesis—Compensatory Recovery Mechanism

**Figure 3:**
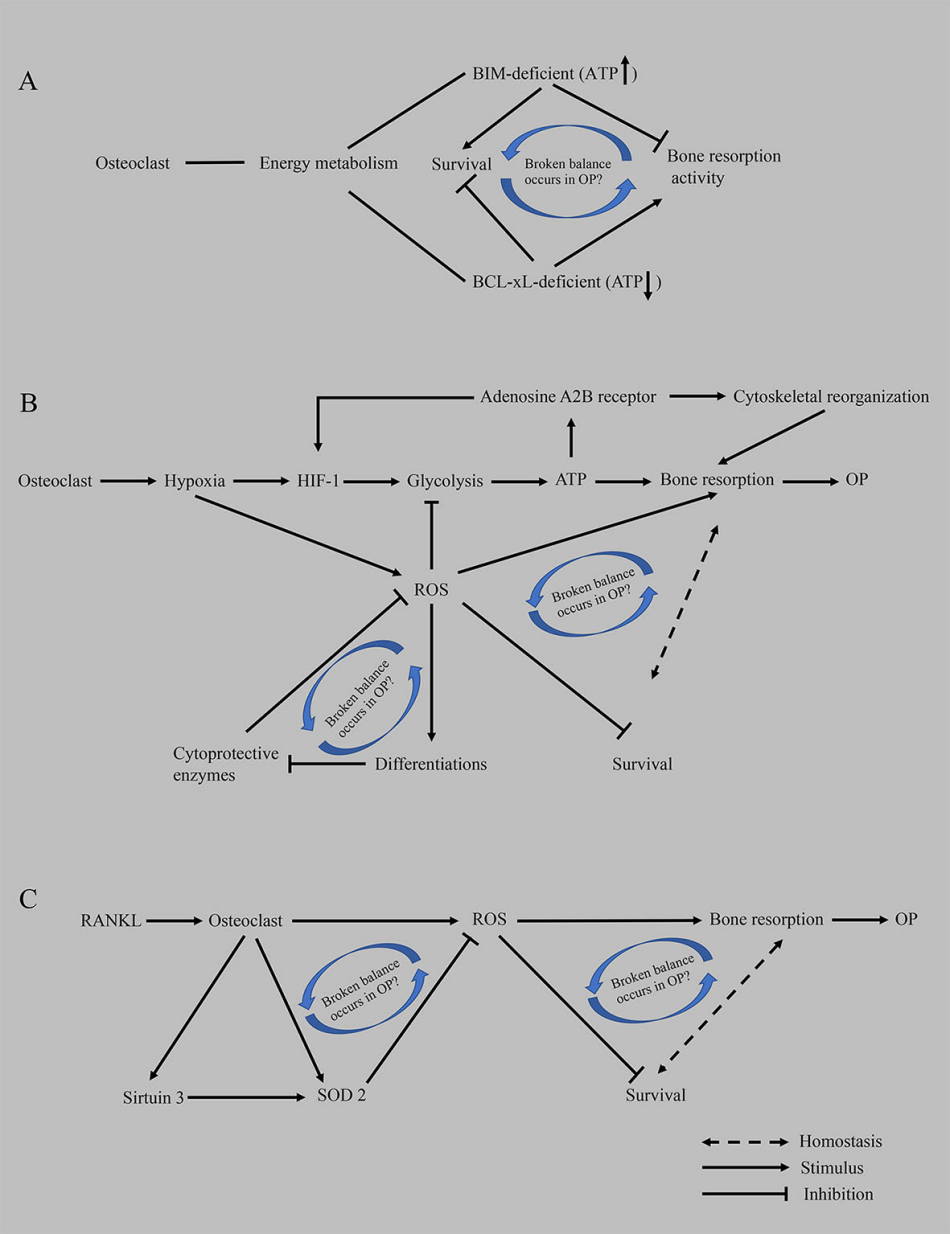
Hypothesis of the compensatory recovery mechanism of osteoclasts. **(A)** Based on proapoptotic BIM and anti-apoptotic BCL-xL, an imbalance between osteoclast survival and bone resorptive capacity occurs in OP. **(B)** The disruption of homeostasis between the enhancement of bone resorption activity mediated by hypoxia and the inhibition of ROS on the survival of osteoclasts promotes the occurrence of osteoporosis. **(C)** RANKL-mediated oxidative stress results in an imbalance between osteoclast survival and bone resorption capacity. ATP, adenosine triphosphate; BCL-xL, B-cell lymphoma-extra large; BIM, BCL-2-like protein 11; HIF-1, hypoxia-induced factor-1; OP, osteoporosis; ROS, reactive oxygen species; RANKL, receptor activator of nuclear factor kappa-B ligand; SOD2, superoxide dismutase 2.

We propose that cell development is a continuous process and that bone remodeling is also a dynamic continuous process. Under physiological conditions, continuous bone resorption by osteoclasts removes old bone to ensure the strength of the bone. Osteoclasts have a bone metabolism homeostasis monitoring mechanism that does not allow excessive bone resorption, such as the differentiation of monocyte/macrophage osteoclasts into mature osteoclasts with strong bone resorption capacity, but they also have reduced viability, a shortened lifespan and increased apoptosis and other characteristics. We infer that this process is a compensatory mechanism. The body induces the differentiation of osteoclasts to increase bone resorption and then relies on an adaptive, local microenvironmental, or systemic adaptation to intervene in osteoclast survival and balance bone resorption. Cells only exert their biological effects when sufficient energy is available at the right time. For example, (1) BIM (a proapoptotic member in BCL-2 family)-deficient osteoclasts show decreased bone resorption activity, despite a prolonged survival time, while BCL-xL (an antiapoptotic member in BCL-2 family)-deficient osteoclasts show increased bone resorption and accelerated apoptosis, implying that the mechanism regulating this inverse correlation remains to be determined (29) (**Figure 3A**); (2) ROS scavenging by cytoprotective enzymes negatively controls the formation of osteoclasts (171, 172) (**Figure 3B**). (3) RANKL increases the expression and activity of SOD2 (an enzyme responsible for reducing superoxide free radicals in mitochondria) and SIRT3 (an NAD-dependent deacetylase responsible for activating SOD2) to negatively regulate osteoclast formation and prevent excessive OC formation in physiological bone metabolism (163) (**Figure 3C**). When osteoporosis occurs and develops, many positive feedback mechanisms are activated in osteoclasts (such as lactate secretion, mitochondrial biogenesis, ROS production, and calcium homeostasis), and many other factors promote the differentiation of osteoclasts; on the other hand, the compensatory regulation of osteoclasts and the whole body also fails. Therefore, in the future, we should devote ourselves to exploring the compensatory mechanism of osteoclasts, which is the mechanism controlling the innate biology of osteoclasts. If this compensatory mechanism can be restored in a targeted manner when osteoporosis develops, the curative effect is presumed to be significant and the side effects will be eliminated instead of blindly stimulating bone formation and inhibiting bone resorption.

## Author Contributions

All authors contributed to the study conception and design. Material preparation, data collection and analysis were performed by WD. The first draft of the manuscript was written by WD. YZ and LT are responsible for monitoring the progress of the entire study and also responsible for the review and revision of manuscript. All authors contributed to the article and approved the submitted version.

## Conflict of Interest

The authors declare that the research was conducted in the absence of any commercial or financial relationships that could be construed as a potential conflict of interest.

## Abbreviations

adenosine triphosphate (ATP); glucose transporter (GLUT1 and GLUT3); lactate dehydrogenase (LDH); monocarboxylate transporters (MCTs); malate dehydrogenase (MDH); matrix metalloproteinases (MMPs); nicotinamide adenine dinucleotide (NADH); nuclear factor of activated T-cells 1 (NFATC1); osteoclast (OC); Osteoporosis (OP); Oxidative phosphorylation (OXPHOS); receptor activator of nuclear factor kappa-B ligand (RANKL); tricarboxylic acid cycle (TCA cycle); T-cell immune regulator 1 (TCIRG1); vacuolar H^+^ adenosine triphosphatase (V-ATPase).
